# The melanocortin receptors as targets for general obesity: contextualizing clinical failures and analyzing future perspectives

**DOI:** 10.3389/fendo.2026.1797586

**Published:** 2026-03-12

**Authors:** Claudia R. Prindle, Thomas L. Bennett, Benjamin H. Rajewski, Kade J. Kelley, Anna C. Impastato, Russell Potterfield, Daniel L. Marks, Jordan Y. Delev

**Affiliations:** Endevica Bio, Northbrook, IL, United States

**Keywords:** food intake, melanocortin (MC), melanocortin-3 receptor, melanocortin-4 receptor, melanocortin-4 receptor agonist, metabolism, obesity, weight loss

## Abstract

The melanocortin system is a genetically conserved and clinically validated target for the treatment of obesity. Hundreds of studies establish the role of the melanocortin receptors, particularly the melanocortin-4 receptor, in food intake, weight control, and energy expenditure. Over the last several decades, the pharmaceutical industry pursued melanocortin-4 receptor-selective agonists for the treatment of general obesity. Many candidates failed to demonstrate meaningful weight loss in the clinic due to potency, selectivity or side effect profiles, leading pharmaceutical companies to dismiss the use of melanocortin compounds for treating general obesity. Recent advancements in the field demonstrate the importance of modulating both melanocortin-3 and -4 receptors for optimal weight loss and offer solutions to mitigate undesired side effects associated with melanocortin agonism. Considering these discoveries, it is imperative to review past clinical attempts and compare them with programs currently in development. A thorough understanding of the shortcomings of past clinical programs will enable the development of novel melanocortin therapeutics for the treatment of general obesity.

## Introduction

**Figure 1:**
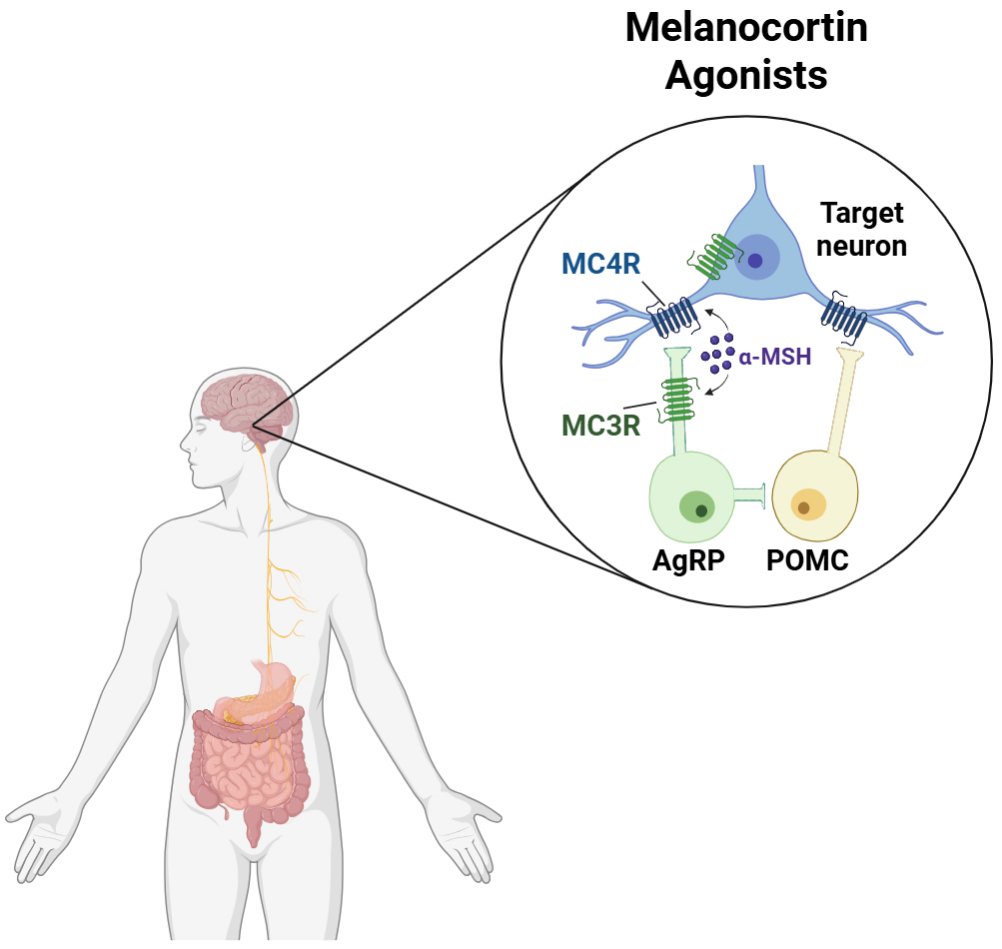
Melanocortin agonists are central regulators of metabolism. At normal physiological levels, endogenous melanocortin agonists (e.g., α-MSH, β-MSH, and γ-MSH) are released by POMC neurons in the arcuate nucleus and centrally regulate metabolism by acting on MC3R and MC4R expressed in the hypothalamus. Created in https://BioRender.com.

The advent of incretin therapeutics revolutionized the standard of care for obesity. With the recent success of amylin receptor agonists, identification of the next key receptor set in obesity is gaining significant attention. The melanocortin (MC) system represents one such target with decades of validation but unrealized clinical potential. The MC system is a family of five G protein-coupled receptors, melanocortin-1 through -5 receptors (MC1R-MC5R), that play an integral role in neuroendocrine signaling (1–4). These receptors are peripherally and centrally expressed in a variety of tissues and organs. MC3R and MC4R are both primarily expressed in the brain as shown in **Figure 1**, particularly in centers of metabolic control including the arcuate nucleus (ARC), paraventricular nucleus (PVN), nucleus of the solitary tract (NTS), and paraventricular thalamus (PVT) (5–8). Historically, MC4R was most heavily implicated in obesity, as its modulation results in changes in food intake, body weight, and energy expenditure (9, 10). Parallel MC4R pathways in different brain regions are responsible for these effects. The ARC-POMC pathway is associated with long-term energy homeostasis and activation of the NTS-POMC pathway is correlated with short-term satiety signals (11–13). Interestingly, POMC neuronal subpopulations in the ARC demonstrate heterogeneity, releasing GABA, glutamate, or both, influencing potential metabolic outcomes (14). MC4R modulation is also linked to effects on blood pressure, glucose metabolism, and sexual stimulation (15–19). Studies show that MC3R influences energy partitioning as well as growth and development (20–23). However, a growing body of evidence suggests that it also affects food intake and body weight (23–25). The other MC receptors are not known to be vital to weight regulation; activation of MC1R increases melanin production while agonism of MC2R and MC5R regulate cortisol and sebum production, respectively (26–28).

The melanocortin receptors are modulated by several endogenous peptides, such as α-, β-, and γ-melanocyte stimulating hormone (MSH) and adrenocorticotropic hormone (ACTH), which are derived from proopiomelanocortin (POMC) precursors (29). MSH peptides centrally activate MC3R and MC4R and act peripherally on MC1R and MC5R (29). In contrast, ACTH is the only endogenous peptide that agonizes MC2R (29). Furthermore, the endogenous peptide Agouti-related peptide (AgRP), which is an inverse agonist on MC4R and a competitive antagonist or ERK-biased agonist on MC3R, is known to increase food intake and body weight (30, 31). The selective activity of these endogenous ligands, as well as the shared central expression of the receptors, suggest that innate co-activation of both MC3R and MC4R plays a major role in regulating metabolic processes.

Early prioritization of MC4R as a therapeutic target for obesity was driven by converging genetic and pharmacological evidence demonstrating its primary role in regulating food intake and body weight. Multiple MC4R-selective ligands advanced to the clinic in the early 2000s (32). Due to narrow therapeutic windows resulting from limited MC3R/MC4R activity, suboptimal pharmacokinetics, and intolerable side effects, none were deemed efficacious in general obesity. In 2020, the FDA approved MC4R-selective agonist setmelanotide for a small patient population of individuals with genetic obesity, considered to be hypersensitive to central MC agonism (33). Consequently, pharmaceutical companies directed much of their current MC agonist development efforts toward identifying and treating MC-agonist deficient states. However, recent breakthroughs in MC biology, such as dual MC3R/MC4R agonism, renewed interest in these receptors as targets for general obesity (25, 34). Combating past failures by increasing MC receptor potency and mitigating side effects will be required to expand MC agonism treatment to general obesity populations.

In this review, we will briefly examine the key discoveries that led to the targeting of MC4R over MC3R for general obesity and discuss the nonclinical and clinical development of melanocortin agonists for the treatment of obesity. We will highlight the successes and failures in the development of clinical MC agonists and detail trends and novel advancements in current melanocortin programs. Finally, we will provide an overview of development considerations for future MC agonists and discuss where this mechanism of action fits into the larger obesity treatment landscape.

## Historical rationale for targeting MC4R

Although both MC3R and MC4R participate in the central regulatory circuitry governing energy homeostasis, MC4R was historically more implicated in metabolic regulation. Early pharmaceutical development efforts, therefore, prioritized selective MC4R agonism over MC3R, a decision guided by rodent studies utilizing genetic data, knockout models, and studies with melanocortin ligands. In 1997, a mouse study demonstrated that genetic ablation of *Mc4r* resulted in an obese phenotype, hyperphagia, and hyperinsulinemia (35). Subsequent studies showed that *Mc3r* knockout mice exhibit increased adiposity without developing overt obesity and, in some cases, display hypophagia (20, 22). These important observations underlie the original hypothesis that MC4R is responsible for weight modulation and food intake, while MC3R plays a more subtle role in energy partitioning. Notably, later work demonstrated that *Mc3r*/*Mc4r* double knockout mice develop a more severe obese phenotype than *Mc4r* knockout animals alone, indicating that the two receptors exert non-redundant effects on energy homeostasis (20).

In line with these observations in rodents, human genetic studies published in the early 2000s identified *MC4R* mutations as the most common monogenic cause of obesity, affecting up to 6% of individuals with severe obesity (36). In contrast, *MC3R* mutations were reported in fewer than 2% of obese individuals (24, 37). Taken together, these distinctions strongly reinforced pharmaceutical strategies focused on selective MC4R targeting.

In parallel with emerging genetic evidence, early pharmacological studies using MC3R-selective ligands, including γ-MSH and synthetic analogues, further shaped receptor prioritization within the MC system (38–41). In rodent models, these ligands failed to reduce food intake and were associated with increases in blood pressure and heart rate (38–41). In contrast, pan-melanocortin agonists such as melanotan-II (MT-II), α-MSH, and β-MSH consistently suppressed food intake (20, 40, 42). Collectively, these findings reinforced the role of MC4R in metabolism while diminishing confidence in MC3R agonism as a viable pathway for weight loss. Consequently, pharmaceutical efforts, likely informed by the prevailing “one drug-one target” bias, prioritized MC4R-selective ligands for the treatment of obesity (43).

While these studies emphasized the role of MC4R in regulating metabolism, later research implicates both MC3R and MC4R in modulation of food intake and body weight. For example, a 2010 study demonstrated that central administration of pan-melanocortin agonist MT-II decreased food intake in WT, *Mc3r* KO, and *Mc4r* KO mice (44). Notably, food intake reduction was not observed in dual knockout mice, further suggesting both receptors are implicated in feeding (44). Consistent with this observation, decreases in food intake and body weight in primates were primarily reported with compounds that exhibit both MC3R and MC4R activity (25, 45, 46). Moreover, in direct comparative studies, dual agonists show greater efficacy than MC4R-selective ligands (25, 34, 47). Importantly, these findings emerged after many MC4R-selective programs had already advanced into the clinic and ultimately failed.

## Previous melanocortin agonist clinical candidates (2004–2022)

In the early 2000s, several MC agonists were developed and trialed as weight management therapies. We limit our discussion in this review to peptides and small molecules that were specifically trialed in a general obesity population between 2004 and 2022. During this era, companies aimed to identify MC4R-specific agonists. While many of these molecules showed promising results in preclinical rodent, minipig, or non-human primate (NHP) models, they could not achieve sufficient therapeutic windows to observe clinical weight loss without adverse events such as cardiac activation (6).

### MK-0493

**Figure 2:**
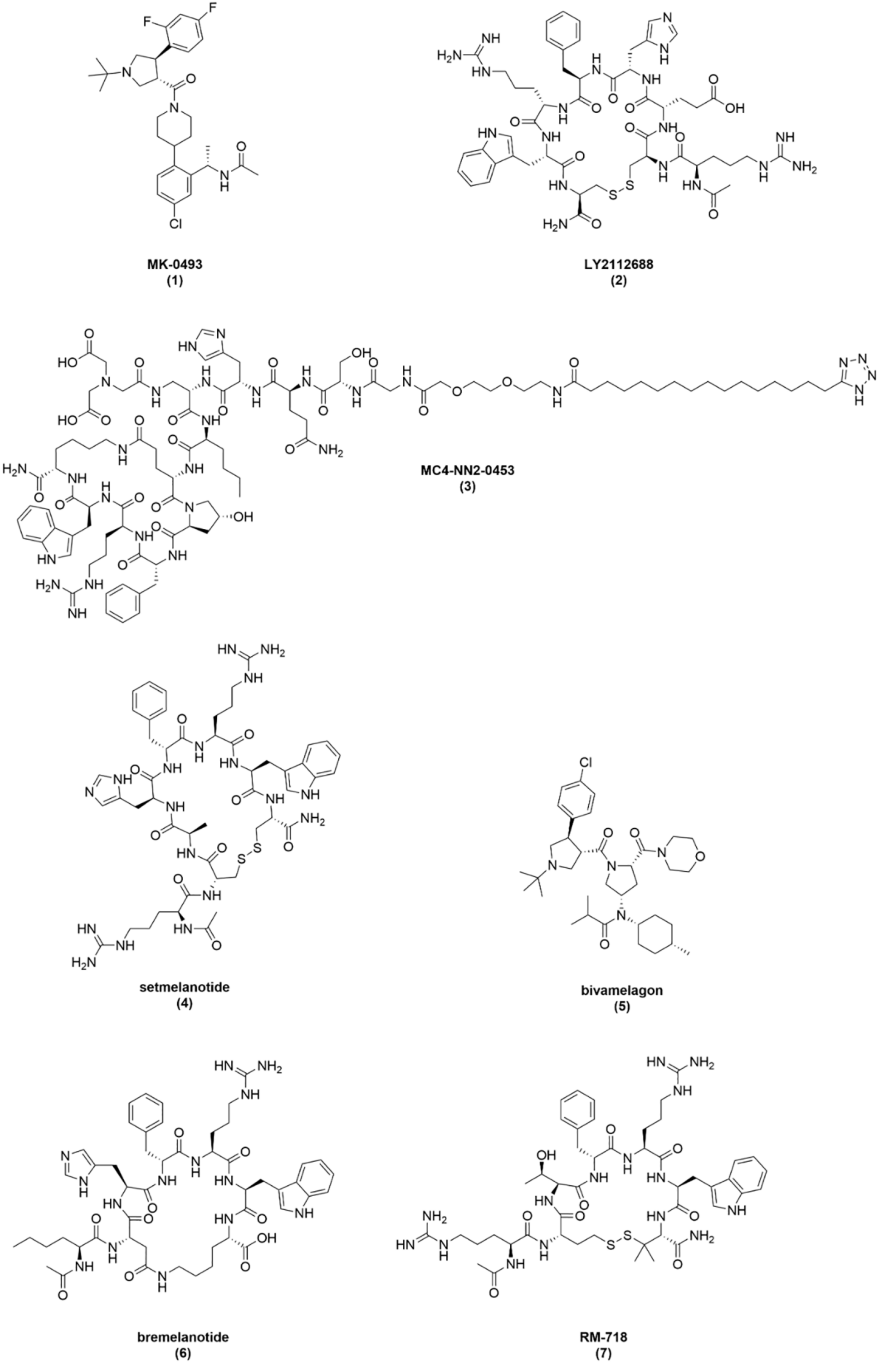
Structures of melanocortin agonist clinical candidates.

MK-0493 is an orally available small molecule developed by Merck (**Figure 2**). It is described as a novel, potent, and selective MC4R agonist, with minimal potency on hMC3R (EC_50_ > 1000 nM) and nanomolar potency on hMC4R (EC_50_ = 7.8 nM, E_max_ = 104%) as shown in **Table 1** (25). While MK-0493 treatment reportedly resulted in reduced energy intake and attenuated weight gain in diet-induced obesity (DIO) rodent models, no publicly disclosed quantitative data to support these findings have been reported (48). Additionally, nonclinical studies in a telemeterized dog model report increases in blood pressure at higher doses (48).

**Table 1:** Potency values, nonclinical highlights, and clinical fates for melanocortin agonists trialed in general obesity.

Compound (description)	Binding, K_i_ (nM)			Activity, cAMP EC_50_ (nM) (E_max_)			Preclinical highlights	Clinical fate	Source
	hMC1R	hMC3R	hMC4R	hMC1R	hMC3R	hMC4R			
MC4-NN2-0453 (lipidated, cyclic peptide)	–	–	–	–	39 (31%)	31 (105%)	34% food intake reduction 48 hours after administration in DIO rats. Compound analog caused 13% loss in body weight in DIO minipigs	Discontinued due to pigment-related AE. Body weight reduction could not be determined	25, 55, 56, 57
	2700	71	0.58	–	–	88 (85%)			
LY2112688 (cyclic peptide)	–	–	–	–	4.2 (91%)	0.048 (100%)	19% decrease in average daily caloric intake in DIO NHPs but caused elevations in blood pressure and heart rate. Reduced body weight of DIO rats by 13 g compared to baseline and 34 g compared to placebo	No clinical efficacy observed. Increases in CV and erectile activity	16, 25, 45, 52
	16.78	56.79	0.55		1.61 (84%)	0.28 (95%)			
	3.99	35.1	1.84	8.12	10.3	0.0857			
AZD2820 (cyclic peptide)	–	9	–	–	no activity	1 (38%)	Reduced body weight rebound of DIO mice after release from weight restriction by 12.4% compared to placebo	Discontinued Allergic reaction SAE	58, 64
setmelanotide (cyclic peptide)	–	–	–	–	0.82 (88%)	0.053 (100%)	Reduced body weight in DIO NHPs by 1.5 kg (~10%) at a dose of 0.5 mg/kg over eight weeks by SC infusion	Approved for genetic obesity	25, 45, 69
	3.9	10	2.1	5.8	5.3	0.27			
MK-0493 (small molecule)	–	–	–	–	>1,000	7.8 (104%)	Reduced food intake and weight gain in DIO rodents	No clinical efficacy observed	25, 48
bivamelagon (small molecule)	–	–	–	–	130 (102%)	0.98 (103%)	Significantly reduced body weight in DIO mice (-11.2%) and rats (-7.4%) over six weeks	Completed Phase 2 Only trialing in patients with hypothalamic obesity	25, 98, 99, 104
	310	–	62	84.82	–	16.28			

During phase I and phase II clinical trials, MK-0493 was generally well-tolerated at both single and multiple doses (**Table 2**) (48, 49). In initial studies, a maximum tolerated dose (MTD) of 400 mg by mouth (PO) once daily (QD) was established in healthy subjects due to dose-limiting side effects such as nausea and vomiting (48). In a follow-on study, general obese participants were administered either placebo or a dose-titrated regimen of MK-0493, in which participants received 400 mg PO QD of MK-0493 for seven days, with dose escalations of 200 mg every seven days until a final dose of 1000 mg PO QD was attained (48). The results of this study demonstrated that a weekly stepped titration approach helped to mitigate dose-limiting side effects and that MK-0493 was generally well tolerated up to the 1000 mg dose (50). In additional clinical studies, MK-0493 was not associated with significant increases in blood pressure or sexual side effects (48, 51).

**Table 2:** Clinical trial analysis of past melanocortin agonists.

Compound	NCT trial #	Cohort size (n)	Trial length (days of dosing)	Dose and RoA	Weight loss from baseline (kg)	Weight loss from baseline (%)	Weight loss vs placebo (kg)	Weight loss vs placebo(%)	Significant weight loss (P<0.05) achieved?	Comments	Source
Setmelanotide	NCT02431442	5	28	0.01 mg/kg, 24 h SC infusion	-3.07	-3.14^a^	-3.97	-4.06^a^	Yes	Cohorts receiving doses ≥ 0.01 mg/kg/24 h reported to also have achieved significant weight loss although values not reported	85, 86
	NCT01749137	33	90	1.0 mg, 24 h SC infusion	-2.2	-2.0	-1.9	-1.7	No		87
	NCT01867437	12	3	1.0 mg, 24 h SC infusion	NA	NA	NA	NA	NA	Significant increase in energy expenditure determined	88, 89
	NCT02041195	8	84	Weeks 1-4: 0.75 mg to 2 mg, BID SC injection Weeks 5-12: 2 mg, QD SC injection	NR	-1.38	NR	-4.27	Yes	–	90
		6	84	Weeks 1-4: 1.5 mg to 2 mg, QD SC injection Weeks 5-12: 2 mg, QD SC injection	NR	-0.74	NR	-3.63	Yes	–	
		10	84	1.5 mg to 2 mg x 12 weeks, QD SC injection	NR	-1.29	NR	-3.57	Yes	–	
		22	84	2 mg, QD SC injection	NR	-2.28	NR	-2.22	Yes	–	
MC4-NN2-0453	–	20	70	0.75, 1.5. 3.0 mg/day, QD SC injection	ND	ND	ND	ND	ND	Only 0.75 mg/day cohort completed the 70-day trial. 1.5 mg/day and 3.0 mg/day cohorts were terminated at 49 and 14 days, respectively, due to pigmentation-related AEs. The effect of MC4-NN2-0453 on body weight and appetite could not be determined	57
LY2112688	–	9-10	7	0.15, 0.45, 1.0 mg/day, 24 h SC infusion	ND	ND	ND	ND	ND	Dose-dependent increases in blood pressure were observed	16
AZD2820	NCT01469923	unknown	14	1.0, 3.0, 6.0 mg, QD SC injection	ND	ND	ND	ND	ND	1 mg and 3 mg cohorts completed the dosing regimen. However, after a single 6 mg dose, one patient experienced a severe allergic reaction resulting in the termination of the trial. No effect on body weight could be determined	62, 63
Bivamelagon	NCT06040372	7	28	10 mg, QD PO	NA	1.3	NA	1.4	No	–	101, 102
		6	28	25 mg, QD PO	NA	-0.8	NA	-0.7	No	–	
		6	28	50 mg, QD PO	NA	-0.6	NA	-0.5	No	–	
		6	28	100 mg, QD PO	NA	0.3	NA	0.4	No	–	
		6	28	200 mg, QD PO	NA	-1.3	NA	-1.2	No	–	
		6	28	400 mg, QD PO	NA	-2.5	NA	-2.4	No	–	
		9	28	600 mg, QD PO	NA	-3.0	NA	-2.9	Yes	–	
MK0493	–	unknown	84	200 mg, QD PO	-1.5^a^	ND	-0.5^a^	ND	No	–	48
		unknown	84	400 mg, QD PO	-2.5^a^	ND	-1.5^a^	ND	No	–	
		unknown	126	QD PO dose escalation strategy: 100 mg × 2 weeks, 200 mg × 2 weeks, 400 mg × 1 week, 600 mg × 1 week, 800 mg × 12 weeks	-2.0^a^	ND	-3.3^a^	ND	No	–	
Bremelanotide	NCT06565611	16	56	Weeks 1-4: 2.5 mg tirzepatide, QW SC injection Week 5-8: 1.25 mg of bremelanotide, QD SC injection	NR	0.2^b^	NR	0.22	No	Bremelanotide was reported to blunt weight regain in participants after cessation of tirzepatide compared to those that crossed over after four weeks from tirzepatide to placebo	126, 127
		49	56	Weeks 1-4: 2.5 mg tirzepatide, QW SC injection Weeks 5-8: 1.25 mg bremelanotide, QD SC injection + 2.5 mg tirzepatide, QW SC injection	NR	-2.1^b^	NR	-2.75	Yes	Combination therapy of low-dose bremelanotide along with low-dose tirzepatide was reported to be synergistic	
	–	27	15	Day 1: 1.25 mg, 1 mg, 1 mg, TID SC injection Day 2-15: 2.5 mg, 2 mg, 2 mg, TID SC injection	-2.1	ND	-1.4^a^	ND	Yes	The trial was conducted exclusively in obese premenopausal women. Significant reduction in caloric intake was also demonstrated	125
		15	4	2.5 mg, 2.0 mg BID SC injection	-1.7	ND	-0.8^a^	ND	Yes		
		15	4	2.5 mg, QD SC injection	>-0.9	ND	NR	ND	No		

^a^Calculated values from reported data.

^b^Baseline value established from Week 4, after tirzepatide dosing.

NR, not reported; ND, not determined; NA, not applicable.

Despite these promising results, MK-0493 did not show clinically significant efficacy in obese human participants at either a 400 mg PO dose or a step-titrated 800 mg PO dose (48). Using an *ad libitum* energy intake model, MK-0493 was associated with a small and marginally significant reduction in total 24-hour energy intake, whereas 30 mg of sibutramine (positive control) was associated with a significant reduction in total 24-hour energy intake (48). Furthermore, MK-0493 resulted in modest weight reduction from baseline, producing small and statistically insignificant effects relative to placebo in a fixed-dose study and after 18 weeks of stepped-titration dosing. These trials led Krishna et al. to conclude an MC4R agonist is not likely to produce clinically meaningful weight loss at well-tolerated doses, and the program was terminated after negative phase II results (48).

While MK-0493 did not achieve clinical success, an analysis of these studies can provide potential rationale for failure. MK-0493 is reported to have low-nanomolar activity on hMC4R (EC_50_ = 7.8 nM, E_max_ = 104%) and no activity on hMC3R (EC_50_ >1000 nM). When compared to other MC4R-selective agonists, MK-0493 is less functionally active on hMC4R. Furthermore, MK-0493 is not active on hMC3R, which is known to play a role in MC-mediated metabolic regulation. These data suggest that a lack of sufficient potency may contribute to minimal efficacy observed in clinical trials.

### LY2112688

LY2112688 was discovered by Eli Lilly through optimization of a scaffold based on the endogenous peptide agonist β-MSH (**Figure 2**). The peptide is a disulfide-cyclized octamer and is reported to have agonist activity on both hMC3R (EC_50_ = 1.61 nM, E_max_ = 84%) and hMC4R (EC_50_ = 0.280 nM, E_max_ = 95%) as shown in **Table 1** (52). Additional pharmacological characterization by our group corroborates its hMC3R potency (EC_50_ = 4.2 nM, E_max_ = 91%) and demonstrates enhanced hMC4R potency (EC_50_ = 0.048 nM, E_max_ = 100%) (25). To determine nonclinical efficacy, LY2112688 was subcutaneously administered to DIO rats at 0.075 and 0.3 μmol/kg once-daily for 14 days (52). Significant weight reductions were reported for high and low doses compared to control over 14 days, although significant food intake reductions were only observed for the first five days of dosing in the high dose cohort. By the final day of dosing, DIO rats administered vehicle gained approximately 20 g of body weight compared to a 3 g increase observed in the 0.075 μmol/kg group. Notably, DIO rats receiving 0.3 μmol/kg of drug lost around 13 g of body weight in 14 days. LY2112688 treatment reportedly resulted in loss of fat mass without changing the lean body mass of DIO rats.

A phase I clinical trial to assess the safety of LY2112688 was conducted in the mid-late 2000s (**Table 2**). In the first arm of the study, five dose cohorts received the peptide at doses of 0.05, 0.15, 0.45, 1.00 and 2.00 mg via 24-hour subcutaneous infusion (16). Increases in blood pressure compared to placebo (4.0 – 8.5 mm Hg) were observed for all dose cohorts beginning three hours after infusion, reaching maximum levels 24 hours after infusion. As a result, the MTD was determined to be 1 mg/day. The second arm of the trial involved three cohorts that received subcutaneous infusions of the peptide at 0.15, 0.45, and 1 mg/day for seven days (16). Observed blood pressure increases from the second arm were sustained across the full trial period. Moreover, the average heart rate of LY2112688-treated participants increased by an average of 3 bpm compared to placebo over seven days. Though the study was too short to observe meaningful body weight changes, significant increases in stretching, yawning, and increased libido/erection were reported. The LY2112688 program was ultimately discontinued due to cardiac side effects (53).

Multiple groups retrospectively studied the efficacy and pressor effects of LY2112688 treatment in nonclinical models. One such study treated telemeterized Göttingen minipigs with twice daily subcutaneous (SC) injections of LY2112688 up to 0.2 mg/kg (54). Minipigs treated with LY2112688 experienced dose-dependent increases in heart rate, blood pressure, and temperature. Doses above 0.1 mg/kg were deemed intolerable, as minipigs demonstrated behaviors such as hunched posture and tremors. Though tolerable, the 0.1 mg/kg dose was 17 times higher than doses administered in Eli Lilly’s clinical trial (54). While there were no comments on changes in food intake or body weight, authors noted that body temperature increases may be due to increased energy expenditure associated with MC agonism. Additionally, a study by Kievit et al. shows that LY2112688 is both efficacious and cardioexcitatory in DIO NHPs (45). The peptide was delivered via SC minipump infusion at doses of 0.17 and 0.5 mg/kg/24 h for seven days. Both low and high doses resulted in food intake reductions of approximately 17% and 14.5%, respectively, but dose-dependence was not observed. However, this efficacy was also accompanied by increases in heart rate (>14 bpm) and blood pressure in both dose groups.

Overall, LY2112688 demonstrated robust nonclinical efficacy across three different species, reducing body weight, food intake, and potentially increasing energy expenditure. Despite these positive results, the peptide induced increases in blood pressure and heart rate that were confirmed in its clinical trial. Ultimately, the program was terminated, most likely due to its cardioexcitatory properties, preventing a full evaluation of its weight loss efficacy. A comparison of LY2112688 and MC-agonist setmelanotide suggests that the cardiac liabilities of LY2112688 are a molecule-specific limitation, rather than a general feature of MC4R agonism. It is possible that differences in receptor selectivity and potency, biased G-protein signaling, systemic and central exposure, or off-target effects are responsible for the different cardiac profiles of these two molecules. LY2112688 also demonstrates enhanced MC3R activity compared to other compounds tested in the late 2000s and early 2010s (**Table 1**). Collectively, the development of LY2112688 underscores the critical need to thoroughly examine cardiac activation in nonclinical studies to develop safe and effective melanocortin agonists.

### MC4-NN2-0453

MC4-NN2–0453 is an MC4R agonist dodecapeptide developed by Novo Nordisk that features a fatty acid acylation moiety for half-life extension as shown in **Figure 2**. The peptide’s development focused on optimizing binding selectivity between MC4R and MC1R to avoid pigmentation side effects associated with MC1R agonism. Though the group reported appreciable 1000:1 selectivity for hMC4R affinity compared to hMC1R affinity, functional cAMP assay results were only reported for hMC4R (EC_50_ = 88 nM, E_max_ = 85%), seen in **Table 1** (55). Our group found MC4-NN2–0453 acts as a full agonist on hMC4R (EC_50_ = 31 nM, E_max_ = 105%) but only had partial agonist activity at hMC3R (EC_50_ = 39 nM, E_max_ = 31%) (25).

Conde-Frieboes et al. examined the nonclinical efficacy of MC4-NN2–0453 in lean rats (55). A 3 mg/kg SC dose of the peptide decreased food intake in rats by 47% percent in the first seven hours following administration and 34% after 48 hours. An analog of MC4-NN2-0453 (MC4-NN1-0182) demonstrated similar food intake decreases in DIO rats and minipigs at 0.3 mg/kg SC, resulting in 7% and 13% loss in body weight, respectively (56). Ultimately, MC4-NN2–0453 was nominated to be the lead clinical candidate due to its improved solubility at physiological pH compared to analog MC4-NN1-0182.

MC4-NN2–0453 entered the clinic in 2010 to investigate safety, tolerability, and pharmacokinetic exposures in overweight and obese participants (**Table 2**) (57). The trial explored subcutaneous single ascending doses (SAD) from 0.03–1.50 mg/kg and multiple ascending doses (MAD) from 0.75–3.0 mg/day. Though all SAD cohorts completed dosing, only one MAD cohort (0.75 mg/day) dosed through 70 days. Two other MAD cohorts (1.5 mg/day and 3.0 mg/day) were halted by the study investigators at 49 and 14 days, respectively, due to skin pigmentation. Because of premature termination of the study, no conclusion was determined regarding MC4-NN2-0453’s effect on body weight.

Skin-related adverse events (AEs) were observed in both the SAD and MAD portions of the trial (57). Formation of melanocytic nevi (45%), including plantar and palmar nevi, and an increase in skin pigmentation (33%) were the most common AEs reported in the MAD portion of the trial. Notably, changes in pigmentation were also reported by participants in two SAD cohorts. Occurrence of this AE after a single dose of MC4-NN2–0453 was likely due to high plasma exposures and an extended half-life imparted by the fatty acid moiety. Dermatological evaluation confirms these skin-related AEs were benign with minimal to mild atypia (57). Further, no melanomas were reported.

Participants on MC4-NN2–0453 also reported sexual arousal, albeit with low incidence (27%) (57). Males experienced longer and more frequent penile erection, without priapism, and females noted changes in sexual arousal. Interestingly, the group reported tolerance with repeated dosing that was independent of exposure levels. While other MC agonists are associated with cardiac side effects, treatment with MC4-NN2–0453 induced no clinically relevant changes in systolic or diastolic blood pressure in the SAD or MAD cohorts.

Despite early termination of the trial, the pharmacokinetics of MC4-NN2–0453 were analyzed for 43 SAD subjects and 48 MAD subjects (57). Plasma concentrations after a single injection of the peptide were dose-dependent and peaked between 50–100 hours. Further, T_1/2_ values averaged between 234 and 274 hours with a plasma clearance of 0.0005 L/h/kg. At 3.0 mg/kg, AUC_0-inf_ was reported to be 2,650,000 h x ng/mL. Due to high stability and high exposures, these parameters suggested the potential for once-weekly administration of MC4-NN2-0453. Dose-dependent exposure was also observed in the MAD portion, with steady-state achieved in 0.75 and 1.5 mg/day subjects on day 29 (57). Additionally, T_1/2_ values were consistent across dose cohorts (232–253 hours) and with the SAD portion. Due to premature termination, steady-state could not be achieved for the 3.0 mg/day cohort and AUC_0-inf_ for both 1.5 mg/day and 3.0 mg/day cohorts could not be estimated.

While exposure levels would indicate potential efficacy, MC4-NN2–0453 treatment did not report weight loss (57). Royalty et al. hypothesize that the brain exposure levels were insufficient to activate the central MC system for weight loss. Coupled with limited potency on MC3R and MC4R compared to other MC agonists, this explanation for the lack of MC4-NN2–0453 efficacy is plausible. Though central exposure may have been low, structural features of the peptide led to long peripheral exposure. The authors suggested that this increased peripheral exposure near the Ki of MC1R may have led to the observed increase in pigmentation. Moreover, the group did not report MC1R functional activity. It is possible that MC4-NN2–0453 is a potent agonist on MC1R despite having lower affinity for the receptor. The authors stress that the trial was terminated to determine the severity of the skin-related AEs and that no participants discontinued the study due to pigmentation effects. This suggests that pigmentation may be a tolerable side effect of MC therapeutics.

### AZD2820

AZD2820 is a cyclic peptide developed by AstraZeneca and Palatin Technologies. Though the structure was not publicly disclosed, AZD2820 was reported to be a partial hMC4R agonist (EC_50_ = 1 nM, E_max_ = 38%) and had no detectable agonist activity on hMC3R as shown in **Table 1** (58, 59). Nonclinical studies detailing the efficacy of AZD2820 are sparse, though a study on body weight reduction and weight rebound in DIO mice was conducted after the clinical trial concluded (58). In this study, DIO mice were either allowed to feed *ad libitum* or were calorically restricted to induce 20% body weight loss prior to 12-week treatment with either 2.64, 10.8, or 53.4 nmol of AZD2820 per day via SC minipump infusion. DIO mice that received the 53.4 nmol dose lost 5.5% body weight, a significant difference compared to the 3.5% gained by the vehicle group. A dose-dependent effect was observed with AZD2820 treatment, though neither intermediate (-1%) or low (+4%) dose groups achieved significant weight loss compared to vehicle. Similarly, only the high dose caused a significant reduction in weight rebound (+10%) compared to vehicle treatment (+22%) when the calorie-restricted mice were allowed to feed *ad libitum*. Reductions in fat mass without concomitant lean mass losses and decreases in plasma leptin and insulin were noted with 53.4 nmol/day doses of AZD2820 (58). Interestingly, no changes in food intake, energy expenditure, or blood glucose were reported. Additionally, erectile activity was observed in all dose groups.

AZD2820 entered a phase 1 clinical trial in 2011 to investigate the safety and tolerability in healthy male volunteers of single ascending doses up to 12 mg, SC QD (**Table 2**) (60, 61). Notably, volunteers receiving 9 and 12 mg doses reported decreases in appetite, however, severe nausea and vomiting as well as increased erections were also reported in these cohorts. AZD2820 did not induce these effects in cohorts receiving 6 mg or less (60, 61). The peptide was administered to overweight healthy participants at 1, 3, or 6 mg as a daily subcutaneous injection for 14 days in a phase 1 MAD trial in 2012 (NCT01469923) (62, 63). Cohorts receiving either 1 mg or 3 mg completed their dosing regimens, but a severe allergic reaction occurred in one patient after a singular 6 mg dose of AZD2820 (63, 64). The participant was hospitalized and the trial was terminated. No changes in blood pressure, body weight, food intake, or trends in erectile activity were observed during the MAD trial (63).

The absence of weight loss coupled with partial agonism on hMC4R suggests that an efficacious dose of AZD2820 was not achieved. This is further evidenced by decreased appetite observed at high, intolerable doses in the phase 1 trial (60, 61). Although the lack of translatability between mouse studies and human clinical trials is confounding, it is possible that AZD2820 may act as a full agonist on mMC4R and only a partial agonist on hMC4R. It is also possible that AZD2820 activates different cell signaling pathways downstream of the MC4R. The reported activity is in relation to the MC4R-G_s_ pathway; however, recent literature suggests that biased activity for the MC4R-G_q_ pathway can also lead to weight loss (65, 66).

### Setmelanotide – Imcivree^®^

Setmelanotide (preclinically known as BIM-22493, RM-493, marketed as Imcivree)™ is the only approved melanocortin agonist for weight loss, but is currently limited to various forms of monogenic obesity. The compound was originally developed by Ipsen but was licensed to Rhythm Pharmaceuticals in 2010 (67, 68). Setmelanotide was approved by the FDA in 2020 for obesity resulting from defects to the melanocortin signaling pathway, such as LEPR, PCSK1, or POMC deficiencies (69). It later gained approval for Bardet-Biedl Syndrome and is currently trialing for indications in hypothalamic obesity (HO) and Prader-Willi Syndrome (70, 71). Although setmelanotide effectively treats these forms of genetic obesity, the compound had mixed results during nonclinical and clinical evaluation for general obesity. While we focus on setmelanotide for general obesity, many other reviews detail its development for genetic obesity (33, 72–77).

Setmelanotide is a MC4R-selective disulfide-cyclized octapeptide that incorporates several noncanonical amino acids (**Figure 2**) (78). The compound has high potency and affinity for hMC4R (EC_50_ = 0.27 nM) with reduced activity on hMC1R (EC_50_ = 5.8 nM) and hMC3R (EC_50_ = 5.8 nM) (**Table 1**) (45). Notably, our group found the compound to be more potent than reported values for both the hMC4R (EC_50_ = 0.053 nM) and the hMC3R (EC_50_ = 0.82 nM) (**Table 2**) (25). Setmelanotide has comparable potency to α-MSH and, like previous endogenous and synthetic melanocortin agonists, signals through the G_s_ pathway (79). Activity on the NFAT signaling pathway of MC4R was also demonstrated, believed to be through the recruitment of G_q_, with superior potencies compared to α-MSH and LY2112688. Early studies using knockout mice confirmed the compound’s weight loss activity is primarily MC4R-mediated (80). However, more recent studies attributed some of its efficacy to MC3R activity as well (25, 34).

Consistent with its approval in various forms of monogenic obesity, setmelanotide demonstrated strong nonclinical and clinical efficacy for genetic obesity. Setmelanotide induced weight loss in *db/db* and *Magel2*-null mice, which mimic leptin resistance and Prader-Willi syndrome, respectively (79, 81). Notably, *Magel2*-null mice were found to be hyperresponsive to setmelanotide compared to wild-type controls, indicating exogenous agonists are more effective in conditions with impaired MC agonist production and downstream signaling (81). Setmelanotide also demonstrated robust efficacy and acceptable safety evaluated in several clinical trials for genetic obesity (33, 75, 77). The compound did not affect blood pressure but caused varying degrees of sexual stimulation and increased pigmentation (82, 83).

Researchers have also evaluated setmelanotide for general obesity. Several nonclinical studies using lean and DIO animal models consistently demonstrated reductions in food intake and body weight (80). Setmelanotide administered to DIO mice at 3.6 μmol/kg via SC injection for 22 days resulted in 24.5% weight loss from baseline (84). In comparison, administration of liraglutide (a GLP-1R agonist) resulted in a 29.6% weight loss from baseline, but when combined with setmelanotide this increased to 35.4% (84). Setmelanotide, both alone and in combination, also demonstrated a significant increase in energy expenditure compared to liraglutide alone. It also preferentially targeted fat mass over lean mass loss when compared to liraglutide or combination therapy.

Setmelanotide also demonstrated positive results in metabolic studies in DIO NHPs. For example, a SC infusion of setmelanotide at 0.5 mg/kg over 24 h resulted in significant food intake reduction (45). This reduction was also significant compared to the same dose of MC4R-selective agonist LY2112688. When the same dose was administered over eight weeks, setmelanotide significantly reduced body weight by 10% compared to baseline with greater loss of body fat compared to lean mass reported (45). This weight loss continued for another two weeks after cessation of treatment, peaking at a mean weight reduction of 13.5% from baseline. By eight weeks after treatment cessation, animals returned to their baseline weight. Although the NHPs continually lost weight during the treatment, food intake was only significantly lower for the first two weeks. Food intake returned to baseline between weeks 4 and 5 and increased above baseline for the remainder of the study, including the weeks following treatment cessation. Therefore, the authors credit other mechanisms besides reduced food intake, like small observed increases in energy expenditure and daytime activity, as additional drivers of weight loss.

Despite impressive nonclinical efficacy, clinical evaluation of setmelanotide for general obesity yielded mixed results, as shown in **Table 2**. In an early phase 1 study of healthy obese participants, setmelanotide demonstrated significant weight loss compared to baseline for all treatment groups receiving ≥ 0.01 mg/kg/24 h via SC infusion (NCT02431442) (85, 86). Quantitative weight loss was only reported for the cohort receiving 0.01 mg/kg/24 h via SC infusion for 28 days, indicating an average weight reduction of 3 kg (-3.1%) compared to baseline and nearly 4 kg (-4.06%) compared to placebo as shown by (**Table 2**). In a phase 2 trial, setmelanotide was dosed at 1 mg/24 h SC infusion over 90 days (NCT01749137) (87). Despite longer duration of treatment, no significant weight loss was observed. In fact, the trial reported a smaller mean weight loss compared to the previous study (**Table 2**). In another trial using the same dose, a significant increase in resting energy expenditure of 6.4% (111 kcal/24 h) was reported (NCT01867437) (**Table 2**) (88, 89).

In addition to SC infusion, a 12-week clinical trial in general obesity was performed with varying doses of setmelanotide given as a SC injection (NCT02041195) (**Table 2**) (90). The initial stages of the trial escalated doses either once or twice daily up to 2 mg/day over four weeks, while the later stage dosed 2 mg/day for the entirety of the trial. Although this trial led to statistically significant weight loss in all cohorts, weight loss was less than 3% from baseline for all treatment groups. Notably, weight loss appeared to be exposure-dependent, with the cohort receiving the maximum dose of 2 mg/day for the entire study exhibiting the greatest weight loss (**Table 2**).

The observed lack of translational efficacy may be explained by subtherapeutic dosing in the clinic for general obesity rather than interspecies differences in melanocortin biology. In nonclinical mouse studies, animals with genetic melanocortin pathway defects were more responsive to setmelanotide than wild-type mice (79, 81). Collet et al. further hypothesized that individuals with upstream mutations to POMC, resulting in reduced amounts of endogenous MC agonists, are more responsive to exogenous agonists compared to those with a functional MC pathway (86). This is corroborated by setmelanotide’s clinical trials, where participants with monogenic obesity or HO respond more strongly to treatment than general obese participants (NCT02041195, NCT01749137) (33, 75, 77, 79, 87, 90–94).

Important discrepancies exist between setmelanotide’s exposure levels in nonclinical studies and clinical trials. In NHPs receiving SC infusions of 0.5 mg/kg/24 h of setmelanotide, the efficacious dose for DIO NHPs determined by Kievit et al., a C_max_ of approximately 150 ng/mL was reached within the first two hours and persisted for the duration of the treatment, resulting in an AUC_24 h_ of 3814 ng x h/mL (45, 95). However, in the clinic, SC infusions of 0.01 mg/kg/24 h resulted in a C_max_ of only approximately 5 ng/mL that was maintained for the entire 28-day study, 30-fold lower than in NHPs (96). A similar lack of exposure was observed in the clinic with subcutaneous injection. A bolus SC dose, currently approved by FDA, of 2 mg of setmelanotide achieved a C_max_ of 27.5 ng/mL with an AUC_24 h_ of 347 ng x h/mL, 10-fold lower than those observed in NHPs (96).

Although it appears that subtherapeutic doses of setmelanotide were used in clinical trials, higher doses of setmelanotide would require careful observation of cardiac side effects. In the clinic, setmelanotide had no overall effects on heart rate and blood pressure at the doses tested (82). Kievit et al. also demonstrated that the compound did not illicit an increase in blood pressure or heart rate in DIO NHPs at the efficacious dose of 0.5 mg/kg/24 h SC infusion over positive control LY2112688, further suggesting cardiac liabilities to be molecule specific (45). However, cardioexcitatory activity was noted in other nonclinical studies. For example, setmelanotide was associated with increased heart rate and blood pressure in rats and Göttingen minipigs (95). These effects were more prominent in rats, more robust after bolus injection, and attenuated after repeated dosing. Setmelanotide was also associated with increases in heart rate followed by a compensatory drop in blood pressure after 0.5 mg/kg bolus SC injections in NHPs (97). Finally, our group demonstrated that SC injections of 3 mg/kg setmelanotide significantly increased nighttime blood pressure in telemeterized NHPs (25). Differences in reported cardioexcitatory effects between SC bolus injection and infusion suggest that these effects are concentration dependent, potentially correlating with a spike in concentration at C_max_. Nonetheless, these findings indicate that setmelanotide, while safe at approved doses, may have a limited therapeutic window at the higher doses necessary to treat general obesity.

The current tolerability profile of setmelanotide in the clinic may also limit the administration of higher doses. Setmelanotide has poor aqueous solubility, but the use of PEG excipients can worsen injection site reactions (34, 78, 82). Setmelanotide is also associated with an increase in skin pigmentation in approximately 69-78% of its approved patient population due to activity on MC1R (**Table 1**) (33, 82). Although this increase is generally considered safe by the FDA and resolves after cessation of treatment, it is still a potential consideration for patient adherence and preference (82). Increased sexual arousal was also reported in both males and females during setmelanotide treatment. These events were generally mild in nature, with 23% of males reporting sexual events without priapism or genital pain (82). Finally, GI-related side effects were reported for setmelanotide, exhibiting dose-dependent incidence (82, 96). These side effects may require significant attention if setmelanotide were to be administered at doses efficacious for general obesity.

In conclusion, setmelanotide is a very effective therapeutic for patients with genetic disruptions in melanocortin signaling pathways, primarily those with low levels of endogenous agonists for this system. Unlike genetic obesity, the peptide only produced limited weight loss in trials for general obesity. A review of the available literature suggests that these results may be due to administration of subtherapeutic doses in the clinic rather than species differences in biology. Additional studies are required to explore this relationship in more detail.

### Bivamelagon

Bivamelagon (formerly LB54640) is an oral MC4R-selective small molecule agonist as shown in **Figure 2**, with nanomolar potency on hMC1R (EC_50_ = 84.82 nM) and hMC3R (EC_50_ = 130 nM) and low nanomolar potency on hMC4R (EC_50_ = 0.98-16.28 nM) (**Table 1**) (25, 98). It was originally developed by LG Chem but was licensed to Rhythm Pharmaceuticals in 2024. Bivamelagon demonstrated nonclinical efficacy in both DIO mice and rats, resulting in statistically significant body weight and food intake reduction when administered at doses ≥10 mg/kg PO (99). For instance, a 10 mg/kg dose significantly reduced body weight by 11.2% and 7.4% in DIO mice and rats, respectively. Additionally, bivamelagon demonstrated an acceptable safety profile in rats and monkeys across IND-enabling toxicology studies (100).

In a phase I clinical trial, bivamelagon was given to obese and overweight participants in SAD and MAD arms of the trial (NCT06040372) (101, 102). Doses up to 600 mg were generally well-tolerated with no clinically significant abnormalities in blood pressure or heart rate, no observed skin pigmentation, and no significant changes to adrenal, urinary or hematology panels. The most frequently reported AEs were mild-to-moderate gastrointestinal events, affecting approximately 41.3% of all participants in the MAD cohorts. Additionally, body weight displayed a dose-dependent reduction of up to 2.9% compared to placebo at the highest dose group (600 mg/kg) for 28 days. These results demonstrate that high doses of bivamelagon were able to produce statistically significant weight loss in a population with general obesity.

Despite statistically significant body weight reduction in phase I, the compound’s selectivity and potency profile may have limited the magnitude of weight loss. Although bivamelagon is reportedly 8x more potent on MC4R than small molecule MK-0493, bivamelagon is still selectively off-MC3R. Notably, dose-dependency was observed, suggesting that higher doses of bivamelagon could achieve even greater weight loss for general obesity. This hypothesis is further supported by the results of bivamelagon’s phase II trial in patients with HO, where bivamelagon was able to achieve BMI reductions comparable to setmelanotide (NCT06046443, NCT07156578) (103, 104). These findings indicate that MC3R activity may play an important role in producing tolerable and clinically meaningful weight loss in a more diverse patient population.

## Current disclosed candidates in nonclinical and clinical development

Recently, chemical advancements of peptides and small molecules and an increased understanding of the melanocortins restored pharmaceutical interest in the development of MC agonists after the underwhelming trials of the early 2000s. In this section we discuss the nonclinical studies and clinical roadmaps of four key therapies that are currently in development: bremelanotide, RM-718, PL7737, and 710GO.

### Bremelanotide — Vyleesi^®^

Bremelanotide (preclinically referred to as bremelanotan or PT-141 and clinically referred to as Vyleesi^®^) is a cyclic heptapeptide that is reported to be a selective MC1R/MC4R agonist (**Figure 2**). Potency values (EC_50_) for bremelanotide were reported by Palatin on hMC1R (EC_50_ = 0.095 nM), hMC3R (EC_50_ = 2.4 nM), and hMC4R (EC_50_ = 4.07 nM) in cAMP assays (105). Our group reported that bremelanotide is less potent on hMC3R (EC_50_ = 5.5 nM, E_max_ = 92%) compared to hMC4R (EC_50_ = 0.22 nM, E_max_ = 99%) (**Table 3**) (25). The lactam-cyclized heptapeptide is based on the structure of MT-II, only differing in structure by the lack of C-terminal amide (106).

**Table 3:** Potency values and nonclinical highlights of melanocortin agonists in development.

Compound (description)	hMC3R EC50 (nM) (Emax)	hMC4R EC50 (nM) (Emax)	Preclinical highlights	Status	Source
Bremelanotide (cyclic peptide)	5.5 (92%)	0.22 (99%)	Reduced food intake in rats	Approved for HSDD Phase 2 for GLP-1 adjuvant	25, 105, 107, 129
	2.4	4.07			
PL7737 (small molecule)	no activity^a^	2 (95%)	Significantly reduced body weight by 5.09% in DIO mice at 30 mg/kg orally over 3 days compared to vehicle	Filing IND 1H26	134, 136, 137
RM-718 (cyclic peptide)	>3,000	1.11	Reduced body weight by 8.4% in DIO rats at 0.5 mg/kg SC over 22 days	Phase 1	130, 131, 132
710GO (cyclic peptide)	0.073 (96%)	0.042 (101%)	Reduced body weight by 11.7% in DIO NHPs at up to 30 mg/kg orally over 13 weeks	Phase 1-ready	25

^a^Language utilized in Obesity Week 2025 poster.

Nonclinically, bremelanotide inhibited food intake in rats (107). Despite this observation, the development program for bremelanotide has largely focused on the treatment of sexual dysfunction, with both nonclinical and clinical studies examining the sexual effects of bremelanotide (NCT00425256, NCT01382719, NCT02333071,NCT02338960, NCT04179734, NCT04943068) (108–120). In 2019, bremelanotide was approved under the brand name Vyleesi for the treatment of hypoactive sexual desire disorder (121). Notably, the main adverse effects of bremelanotide include increases in systolic and diastolic blood pressure, nausea, vomiting, flushing, headache, and an increase in skin pigmentation (122–124).

More recently, bremelanotide was evaluated for its potential applications in metabolic disorders. In two phase I trials, the effect of bremelanotide on body weight was assessed in obese women (**Table 2**) (125). Study A matched participants 1:1 to receive either placebo or bremelanotide subcutaneously three times daily for 15 days (1.25 mg/1.00 mg/1.00 mg on day 1 and 2.5 mg/2.0 mg/2.0 mg on following days), and Study B assigned participants to receive either once-a-day (2.5 mg) or twice-a-day (2.5 mg/2.0 mg) doses of bremelanotide versus placebo for four days. Pharmacokinetic analysis indicated a rapid increase of bremelanotide concentration after SC administration, but no accumulation was observed. Consistent with nonclinical observations, transient and minimal changes in blood pressure were observed for one hour following administration. The most frequent adverse events reported were injection site reactions, an increase in skin pigmentation, and nausea. In Study A, participants receiving bremelanotide exhibited significantly greater reduction in body weight compared to placebo and reduction of mean caloric intake of approximately 400 kcal/day compared to placebo over 16 days (**Table 2**). In Study B, participants receiving bremelanotide twice daily exhibited significantly greater reduction in body weight compared to placebo (-1.7 kg vs -0.9 kg, respectively) and a reduction in total caloric intake of between 398–469 kcal compared to placebo over four days.

Furthermore, a phase II trial assessed bremelanotide as a GLP-1 adjuvant to tirzepatide for the treatment of obesity (NCT06565611) (**Table 2**) (126, 127). In these studies, obese participants received 2.5 mg tirzepatide SC QWK for four weeks, followed by four weeks of 2.5 mg tirzepatide SC QWK and/or 1.25 mg bremelanotide SC QD, and finally four additional weeks without any treatment. The results of this trial show that both drugs alone and in combination demonstrate improvements in appetite suppression, fullness, and satiety. Bremelanotide matched or exceeded tirzepatide in appetite suppression, and low-dose bremelanotide helped prevent weight rebound after tirzepatide cessation (128). Moreover, the mean percent change in body weight from baseline at week 8 was significant for participants receiving tirzepatide alone (-1.9%) and in combination with bremelanotide (-2.8%) compared to placebo groups (129). These findings suggest that the addition of low-dose MC4R agonist can be used as a safe and potentially effective strategy for achieving and maintaining weight loss when combined with incretins.

### RM-718

RM-718 is a cyclic heptapeptide developed by Rhythm Pharmaceuticals using setmelanotide as a template, with a focus on increasing MC4R potency and selectivity while limiting activity on MC1R to reduce skin pigmentation effects for once-weekly injection (**Figure 2**) (130). It is a potent and selective MC4R agonist, with low nanomolar activity on hMC4R (EC_50_ = 1.1 nM) and minimal activity on hMC1R (EC_50_ = 516.38 nM) and hMC3R (EC_50_ > 3,000 nM) (**Table 3**) (130, 131).

RM-718 has undergone nonclinical evaluation in various rodent models. In DIO rats, RM-718 demonstrated comparable activity to setmelanotide on weight and food intake reduction in a 14-day study (130). Compared to vehicle, administration of 0.5 mg/kg SC of RM-718 produced a significant reduction in food intake and a decrease of 8.4% in body weight. In a telemeterized NHP study, RM-718 caused no significant changes to mean systolic blood pressure and mean diastolic blood pressure over the course of a 3-day SC infusion (1 and 5 mg/kg) compared to 2 days pre-dose (130). While data was not shown, Rhythm confirmed cardiovascular safety with RM-718 with doses up to 30 mg/kg SC, QW, thereby providing a >30-fold safety margin (130).

Currently, RM-718 is being tested in a four-part phase I clinical trial in participants with general obesity, HO, and Prader-Willi Syndrome (NCT06239116). The first three parts (Part A, B, C) are completed (130, 132). Part A is an assessment of single ascending doses from 3–50 mg SC QW, Part B is an assessment of four multiple ascending doses from 3–40 mg SC QW, and Part C is an open label assessment of four multiple ascending doses from 10–40 mg SC QW. Part D of the trial is ongoing and will evaluate the effect of weekly injection of RM-718 in 20 participants with Prader-Willi syndrome over the course of 26 weeks (133).

### PL7737

PL7737 is a MC4R agonist small molecule in development by Palatin Technologies for multiple obesity indications. In addition to a reported focus on labeling in hypothalamic obesity, PL7737 was granted Orphan Drug Designation status by the U.S. Food and Drug Administration for a rare form of genetic obesity caused by leptin receptor deficiency (134, 135).

While the compound’s structure is undisclosed, it is described to have potent activity on hMC4R in a functional cAMP assay (EC_50_ = 2 nM, E_max_ = 95%), inactivity on hMC3R, and reduced potency on hMC1R (25, 136). PL7737 demonstrated nonclinical efficacy in DIO mouse and DIO rat models. In DIO mice, PL7737 achieved significant weight reductions after just one day of treatment when orally administered once-daily at 10 and 30 mg/kg (137). When administered at 3 mg/kg, significant body weight reduction occurred after three days. No acute or chronic effects on systolic blood pressure were observed in DIO mice receiving twice-daily oral PL7737 at 20 mg/kg for two weeks, suggesting the compound lacks the cardiac effects observed in other melanocortin compounds (136). To confirm the metabolic effects are on-target, PL7737 was administered twice-daily to DIO and *Mc4r* KO mice at 30 mg/kg over five days (137). While *Mc4r* KO mice treated with PL7737 showed no significant decline in food intake or weight, DIO mice treated with PL7737 demonstrated significantly reduced food intake and significantly reduced body weight by 7.06% compared to the vehicle group, confirming the metabolic effects of PL7737 require intact MC4R signaling.

In DIO rats, oral PL7737 produced significant weight loss after four days of treatment (138). Combining a high dose of oral PL7737 with dual GIPR/GLP-1R agonist tirzepatide caused DIO rats to lose 15% of their body weight compared to vehicle control after four days of treatment, an additive effect compared to either treatment alone (138). PL7737 similarly demonstrated safety in rats, clearing a 28-day non-GLP toxicity study and demonstrating no erectogenic activity after subcutaneous dosing of 3 and 10 mg/kg/day (136, 137). Finally, PL7737 demonstrated no hERG activation or mutagenic potential in cell-based functional assays (136). IND-enabling toxicology studies are still ongoing, with an IND filing and a phase 1 SAD/MAD trial expected in the first half of 2026. It is unclear whether the compound will be trialed in general obesity due to its focus on hypothalamic obesity.

### 710GO

710GO is an orally available cyclic peptide discovered by Endevica Bio and under development by Kalohexis. The compound is a potent dual agonist on hMC3R (EC_50_ = 0.073 nM, E_max_ = 96%) and hMC4R (EC_50_ = 0.042 nM, E_max_ = 101%) (25). It was designed to reproduce the activity of the endogenous agonist α-MSH, which naturally signals via both MC3R and MC4R to achieve weight loss. Near equipotent activity on both receptors differentiates 710GO from previous melanocortin therapeutics that were designed to selectively target MC4R.

This novel approach to melanocortin receptor selectivity for weight loss was informed by our previous work demonstrating that co-administration of an MC4R-selective agonist and an MC3R-selective agonist caused a greater reduction in food intake and body weight in DIO NHPs than administration of either compound alone, yielding 7.8% weight loss after 36 days (25). In this study, we hypothesized that MC3R plays a role in metabolic boundary control, and that activating MC3R is necessary to surpass established homeostatic set points and potentiate more profound weight loss.

The role of dual agonism is further emphasized by 710GO, which displays agonist activity on both MC3R and MC4R. 710GO demonstrated nonclinical efficacy in DIO rats and NHPs (25). After daily oral administration in DIO NHPs, 710GO significantly reduced body weight by 11.7% compared to vehicle over 13 weeks. Furthermore, compared to weight rebound observed with GLP-1 treatments, weight loss caused by 710GO was more enduring. Two weeks after cessation of treatment, NHPs treated with 710GO had not begun to regain any weight (25). Instead, 710GO-treated NHPs lost an additional 0.39% of their initial body weight during these two weeks. By six weeks after treatment cessation, they had only regained 34.2% of the total weight lost during treatment. This slope of weight regain was not significantly different when compared to animals receiving placebo. Comparatively, NHPs treated with liraglutide for 43 days had regained more than 100% of their total weight lost in the two weeks following treatment cessation (139). In further contrast to GLP-1 therapies, which can result in up to 60% lean mass loss as a proportion of total weight lost, 710GO primarily targets fat mass (140). Lean mass only accounted for 9.9% of total mass lost after thirty-five days in 710GO-treated DIO NHPs (25).

The therapeutic value of 710GO as a GLP-1 adjuvant was also evaluated. Over 19 days, 710GO was administered as an adjuvant with semaglutide to DIO NHPs, causing 7.6% weight loss compared to 4.1% when semaglutide was administered alone (25). This increase in weight loss with the combination regimen was also found to be statistically significant over semaglutide alone. Combination therapies of melanocortins and incretins could enable similar metabolic effects with lower respective doses and exposures, mitigating off-target side effects without compromising weight loss activity. As evidence of this, at the chosen doses, the combination therapy did not induce GLP-1-associated vomiting or GI distress in the animals, despite causing greater weight loss than semaglutide alone. In addition, reduced weight regain and preservation of lean mass makes 710GO a promising candidate for combination therapies with GLP-1s (25).

Importantly, 710GO does not demonstrate the cardiac liabilities observed with other melanocortin agonists. At doses six times greater than its efficacious dose (60 mg/kg, PO), 710GO did not cause changes in telemeterized NHPs to any of the measured cardiac parameters, including systolic blood pressure, diastolic blood pressure, heart rate, and corrected QT interval (25). We attributed the cardiac effects of other melanocortin agonists to off-target activation; reportedly, the C-terminal extension of 710GO improves specificity for the melanocortin receptors while mitigating off-target binding. Additionally, dual agonism of MC3R and MC4R enables greater weight loss at lower doses, widening the therapeutic window between efficacy and potential off-target effects. These preclinical findings suggest that 710GO may address key translational challenges, combining robust metabolic efficacy with an improved cardiac safety profile in NHPs. However, clinical evaluation will be necessary to confirm these properties in humans.

## Future directions

The melanocortin system is well established as a key regulator of metabolic processes, leading many pharmaceutical and biotech companies to target these receptors, particularly MC4R, for their anti-obesity potential. These receptors are highly conserved and robustly characterized, as genetic knockouts in nonclinical species and human mutations result in similar obese phenotypes (20, 22, 23, 35, 36). However, MC4R agonists generally demonstrated a significant drop in efficacy between preclinical and clinical studies. Since setmelanotide and bivamelagon achieved dose-dependent weight loss in general obesity, target invalidation is an unlikely explanation for this discrepancy (NCT02041195, NCT02431442, NCT06040372) (85, 90, 101). Instead, the magnitude of clinical benefit is likely constrained by therapeutic window, limiting the extent to which the melanocortin pathway can safely be engaged.

Previous melanocortin agonists that failed clinical trials for general obesity share common features that constrain their therapeutic windows, including low receptor potency, poor safety profiles, and suboptimal pharmacokinetics. Because melanocortin modulation of energy homeostasis is centrally mediated, drug developers must overcome large pharmacokinetic hurdles for CNS penetration (6, 53, 141). For instance, MC4-NN2-0453, which did not demonstrate clinical efficacy, achieved substantial peripheral circulation but may have had limited central penetrance. In contrast, limited solubility may have restricted the potential efficacy of setmelanotide by hampering its systemic exposure.

Although high peripheral exposure may be required to obtain the fractional central exposure necessary to illicit a pharmacologic response, insufficient selectivity between MC1R and MC4R activity can lead to peripheral activation of MC1R (26, 55, 57). This was observed with MC4-NN2–0453 which caused pigmentation-related AEs in the clinic as a result of its strong MC1R agonism from high sustained systemic exposures (26, 55, 57). While it is important to note that skin pigmentation could be considered objectionable by certain populations, it is not normally considered one of the SAEs outlined by the FDA and ICH E2A/E6 guidelines (142, 143). Although 78% of participants receiving setmelanotide on trial showed signs of increased skin pigmentation, it ultimately received FDA approval in 2020 (144). Since then, few patients discontinued setmelanotide due to objections over pigment changes; these changes quickly reached a clinically acceptable plateau and were not progressive over five years of clinical exposure (145). Further, MC1R-related pigmentation is considered safe, leading researchers to pursue it for UV-protective therapeutics (146–149).

Increases in sympathetic outflow remain the largest safety concern for melanocortin agonists after compounds such as LY2112688 and bremelanotide demonstrated increases in both blood pressure and heart rate in clinical trials (16, 150). Previously, these pressor effects were primarily associated with MC4R activation (15, 151). However, other MC4R-selective agonists demonstrated no pressor effects in the clinic (48, 57, 60, 62, 82, 101). Nonclinical cardiac studies have variable results depending on species, dose, route of administration, and length of dosing, further indicating that the mechanism for cardiac activation may be more complex than previously believed.

There are several non-mutually exclusive mechanisms to rationalize the cardiac effects observed with melanocortin agonism. One hypothesis posits that pressor effects are mediated by the activation of central MC receptors but not peripheral, supported by observed cardiac effects after central α-MSH administration but not when α-MSH is delivered intravenously (152). This implies that peripherally restricted melanocortin agonists may not alter blood pressure. However, several melanocortin agonists demonstrated activation of central MC receptors or penetration into the CNS without noted cardiac side effects (25, 98, 102). A second mechanism suggests that biased agonism, such as activity on the MC4R-G_q_ pathway, may mitigate pressor effects while maintaining anorectic activity (66, 153). Although some emerging data support this hypothesis, the pharmacological profile of many melanocortin agonists that do not induce pressor effects remain incompletely characterized. Finally, a third hypothesis postulates that off-target effects may be responsible for cardioexcitatory activity (154). In nonclinical studies, the C-terminal Arg-Phe-Xaa peptidic motif common to RF-amide receptor ligands is associated with blood pressure and heart rate changes (152, 155–158). A related motif is found within the melanocortin pharmacophore that is incorporated into the design of many MC4R-selective agonists. Extension or disruption of this motif is proposed to ameliorate associated pressor effects but the specific receptor or receptors mediating these effects were not identified (25).

It is important to note that MC4R potency correlates well with observed clinical weight loss. Compounds such as MK-0493 and MC4-NN2-0453, which do not activate MC4R as potently as other agonists, demonstrated no weight loss in humans despite dosing for 126 and 70 days, respectively (48, 49, 57). Conversely, compounds with improved MC4R potency caused significant weight loss during their trials, including setmelanotide and, in some studies, bivamelagon (85, 90, 101). However, MC4R agonism alone may be insufficient to widen the therapeutic window for melanocortins to prove efficacious in general obesity. Reported nonclinical studies suggest that dual agonism of MC3R and MC4R offers increased efficacy without triggering side effects (25). LY2112688 and setmelanotide demonstrate comparable MC4R activity; however, setmelanotide is considerably more potent on MC3R and did not result in cardiac activation in DIO NHPs in a head-to-head experiment (45). The deliberate targeting of both MC3R and MC4R ultimately culminated in the first disclosed equipotent dual agonist, 710GO. This compound, based on the endogenous dual agonist α-MSH, demonstrated increased MC3R activity compared to setmelanotide and significant body weight reductions in DIO NHPs (25).

Current melanocortin agonists are addressing some of the issues encountered by previous failed agonists. RM-718, PL7737 and 710GO all report efficacy with acceptable nonclinical safety profiles, including no evidence of cardiac activation (25, 130). RM-718 and PL7737 are also selective away from MC1R and should mitigate increases in skin pigmentation. The route of administration is another focus of future melanocortin agonist development, as the standard of care for obesity has moved beyond daily injectable administration. PL7737 and 710GO emphasize oral delivery while RM-718 aims towards being a once-weekly MC4R-selective injectable (25, 130, 132, 137, 138). Despite these advancements, both Rhythm Pharmaceuticals and Palatin Technologies report focusing their agonist programs on treating monogenic or syndromic obesity populations (130, 134). PL7737 has comparable potency to small molecule agonists (MK-0493, bivamelagon) that were inefficacious in general obese populations and RM-718 is less potent on MC3R and MC4R than setmelanotide, raising concerns for potential lack of efficacy in future general obesity trials.

Incretins, the current standard of care for obesity, have drawbacks including GI-side effects, rebound after cessation of treatment, and lean mass loss (140, 159, 160). Melanocortin agonism has been associated with body composition outcomes that differ from those typically observed with incretin-based therapies, including preferential reductions in fat mass and more limited weight rebound (25, 52, 58, 128). Further, disruption of the melanocortin system has been linked to changes in food preference, particularly towards carbohydrate-dense and high-fat foods (161, 162). Co-activation of both systems could mitigate GLP-1 drawbacks while having an additive effect on weight loss, allowing for lower doses of each therapy. Recently, clinical evaluation of bremelanotide as an adjuvant shows that combination with tirzepatide improved appetite suppression, fullness, and satiety when compared to tirzepatide alone (126–129). Combination treatment did not result in blood pressure increases or GI-side effects, which were observed when bremelanotide and tirzepatide were administered as monotherapies, respectively (126–129). A low maintenance dose of bremelanotide following tirzepatide cessation also appears to prevent significant body weight regain seen with tirzepatide (126–129). Furthermore, PL7737 and 710GO also demonstrated efficacious combination with GLP-1s, albeit nonclinically (25, 130, 137). As GLP-1 adjuvants, MC4R-selective agonists are positioned as valuable therapeutics for both general and genetic obesity.

While current clinical candidates focus primarily on selective MC4R agonism for the limited treatment of genetic and hypothalamic obesity, 710GO appears to be the only current developmental program poised to address general obesity as a monotherapy. Promising nonclinical evidence suggests that equipotent MC3R agonism can improve the therapeutic window between weight loss and cardiac activation, suggesting 710GO’s potential as a monotherapy beyond its demonstrated success in combination with semaglutide (25). 710GO also demonstrated preferential fat mass loss, consistent with observations reported for LY2112688 and setmelanotide (25, 52). Coupled with reported mitigation of rebound, MC3R/MC4R agonism may result in healthier weight loss than GLP-1s. Improved translatability to humans will remain unconfirmed, however, until 710GO enters the clinic.

Key limitations of GLP-1 therapies have driven the swift development of differentiated assets for the treatment of obesity. The melanocortin system, as the central mediator of energy homeostasis in humans, is receiving renewed interest and holds untapped potential as a target for obesity therapeutics. We have reviewed the failures of previous clinical candidates, outlining an updated set of criteria that define successful melanocortin agonists for obesity: high potency, preferred selectivity profiles, PK exposures that enable central penetrance, and clean cardiac safety profiles. While melanocortin agonists have historically failed to meet these criteria, limiting their use to genetic obesity, emerging melanocortins show promise as both monotherapies and GLP-1 adjuvants for general obesity patients. Additionally, co-agonism of MC4R and MC3R may enable 3^rd^-generation melanocortin agonists to access a wider patient population in general obesity.
